# Supplementary material for: Association between housing tenure and self-rated health in Japan: Findings from a nationwide cross-sectional survey
Source: PLoS One. 2019 Nov 14;14(11):e0224821. doi: 10.1371/journal.pone.0224821 (PMC6855483; doi:10.1371/journal.pone.0224821)
Supplement: S1 Table — (DOCX) [file pone.0224821.s001.docx]

**S1 Table.** Basic attributes of individuals included in this study and those excluded from analyses

|  |  | Potential individuals^a^ | Included individuals^b^ | Excluded individuals^c^ | *P*-value^d^ |
| --- | --- | --- | --- | --- | --- |
|  |  | (n = 73,740) | (n = 59,784) | (n = 13,956) |  |
|  |  | % (n) | % (n) | % (n) |  |
| Age^e^ | |  |  |  | <0.001 |
|  | 20–44 years | 37.0 (27,291) | 39.3 (23,512) | 27.1 (3,779) |  |
|  | 45–64 years | 35.8 (26,396) | 36.6 (21,869) | 32.4 (4,527) |  |
|  | ≥65 years | 27.2 (20,053) | 24.1 (14,403) | 40.5 (5,650) |  |
| Gender | |  |  |  | 0.288 |
|  | Men | 47.8 (35,257) | 47.9 (28,641) | 47.4 (6,616) |  |
|  | Women | 52.2 (38,483) | 52.1 (31,143) | 52.6 (7,340) |  |

^a^ Individuals aged 20 or over who had neither been admitted to hospital nor had activities of daily living (ADL) disability at survey

^b^ Individuals with complete data on age, hospital admission, ADL, housing tenure (HT), household crowding, and self-rated health (SRH).

^c^ Individuals who had missing information concerning age, hospital admission, ADL, HT, household crowding, and/or SRH.

^d^ Differences between included and excluded individuals were analyzed using the Chi-squared test.

^e^ Persons with missing data for age (n = 111) were excluded from this analysis.
