# Supplementary material for: Association between housing tenure and self-rated health in Japan: Findings from a nationwide cross-sectional survey
Source: PLoS One. 2019 Nov 14;14(11):e0224821. doi: 10.1371/journal.pone.0224821 (PMC6855483; doi:10.1371/journal.pone.0224821)
Supplement: S2 Table — (DOCX) [file pone.0224821.s002.docx]

**S2 Table.** Characteristics of study participants

|  |  | % | (n) | Poor SRH (%) |
| --- | --- | --- | --- | --- |
| Age | 20–34 years | 20.6 | (12,311) | 8.5 |
|  | 35–49 years | 27.4 | (16,367) | 11.4 |
|  | 50–64 years | 27.9 | (16,703) | 14.6 |
|  | 65–74 years | 14.1 | (8,445) | 19.3 |
|  | ≥75 years | 10.0 | (5,958) | 29.9 |
| Gender | Male | 47.9 | (28,641) | 13.7 |
|  | Female | 52.1 | (31,143) | 15.5 |
| Marital status | Married | 67.4 | (40,296) | 14.4 |
|  | Never-married | 20.7 | (12,351) | 10.8 |
|  | Widowed/divorced | 11.9 | (7,137) | 22.6 |
| Family size | ≥5 | 16.0 | (9,579) | 12.3 |
|  | 3–4 | 47.7 | (28,540) | 13.1 |
|  | 2 | 25.5 | (15,273) | 17.5 |
|  | 1: living alone | 10.7 | (6,392) | 18.1 |
| Smoking status | Non-smokers | 73.8 | (44,097) | 14.9 |
|  | Current smokers | 23.6 | (14,090) | 13.5 |
|  | Missing | 2.7 | (1,597) | 17.7 |
| Chronic medical conditions^a^ | Absent | 57.2 | (34,203) | 6.6 |
|  | Present | 41.3 | (24,683) | 25.8 |
|  | Missing | 1.5 | (898) | 13.0 |
| Education (years of schooling) | University (≥13 years) | 38.5 | (23,027) | 11.2 |
|  | High school (10–12 years) | 38.8 | (23,223) | 14.8 |
|  | Junior high school (<10 years) | 13.7 | (8,192) | 23.4 |
|  | Missing | 8.9 | (5,342) | 15.4 |
| EHE (Japanese thousand yen) | High: upper tertile (>161) | 29.3 | (17,508) | 14.5 |
|  | Moderate: middle tertile (106-161) | 32.4 | (19,342) | 14.5 |
|  | Low: lower tertile (<106) | 34.3 | (20,512) | 14.7 |
|  | Missing | 4.1 | (2,422) | 16.2 |
| Occupation | Upper non-manual | 19.4 | (11,606) | 10.8 |
|  | Lower non-manual | 24.8 | (14,844) | 10.9 |
|  | Manual | 14.7 | (8,808) | 11.6 |
|  | Non-working | 34.2 | (20,465) | 21.1 |
|  | Missing | 6.8 | (4,061) | 13.7 |
| Household crowding | Not crowded | 53.0 | (31,694) | 16.4 |
|  | Crowded | 47.0 | (28,090) | 12.7 |

EHE, equivalent household expenditures per month; SRH, self-rated health.

^a^ Chronic medical conditions included hypertension, diabetes mellitus, cerebrovascular disease, heart disease, and cancer.
