# Supplementary material for: Association between housing tenure and self-rated health in Japan: Findings from a nationwide cross-sectional survey
Source: PLoS One. 2019 Nov 14;14(11):e0224821. doi: 10.1371/journal.pone.0224821 (PMC6855483; doi:10.1371/journal.pone.0224821)
Supplement: S3 Table — (DOCX) [file pone.0224821.s003.docx]

**S3 Table.** Adjusted odds ratios for poor self-rated health based on stratified analyses by age, gender, and chronic medical conditions

|  |  | n | OR (95% CI) | *P*-value | n | OR (95% CI) | *P*-value | n | OR (95% CI) | *P*-value |
| --- | --- | --- | --- | --- | --- | --- | --- | --- | --- | --- |
| Age^a^ | | 20–44 years (n = 23,512) | | | 45–64 years (n = 21,869) | | | ≥65 years (n = 14,403) | | |
|  | Owner-occupied | 14,862 | 1.00 |  | 17,823 | 1.00 |  | 12,669 | 1.00 |  |
|  | Privately rented | 5,384 | 1.21 (1.07-1.37) | 0.003 | 2,202 | 1.51 (1.32-1.71) | <0.001 | 837 | 1.28 (1.08-1.52) | 0.005 |
|  | Provided housing | 1,022 | 1.13 (0.90-1.42) | 0.300 | 399 | 1.01 (0.74-1.37) | 0.958 | 40 | 1.01 (0.47-2.20) | 0.978 |
|  | Publically subsidized | 1,212 | 1.04 (0.85-1.28) | 0.702 | 884 | 1.54 (1.29-1.85) | <0.001 | 637 | 1.35 (1.12-1.63) | 0.002 |
|  | Rented rooms | 1,032 | 1.24 (0.99-1.54) | 0.058 | 561 | 1.57 (1.24-1.98) | <0.001 | 220 | 1.33 (0.98-1.82) | 0.071 |
| Gender^b^ | | Men (n = 28,641) | | | Women (n = 31,143) | | |  |  |  |
|  | Owner-occupied | 21,480 | 1.00 |  | 23,874 | 1.00 |  |  |  |  |
|  | Privately rented | 4,237 | 1.39 (1.24-1.56) | <0.001 | 4,186 | 1.32 (1.19-1.46) | <0.001 |  |  |  |
|  | Provided housing | 816 | 1.15 (0.89-1.48) | 0.285 | 645 | 1.11 (0.87-1.42) | 0.407 |  |  |  |
|  | Publically subsidized | 1,221 | 1.41 (1.19-1.66) | <0.001 | 1,512 | 1.24 (1.07-1.44) | 0.004 |  |  |  |
|  | Rented rooms | 887 | 1.18 (0.95-1.47) | 0.140 | 926 | 1.57 (1.31-1.89) | <0.001 |  |  |  |
| Chronic medical conditions^c^ | | Absent (n = 34,203) | | | Present (n = 24,683) | | |  |  |  |
|  | Owner-occupied | 24,573 | 1.00 |  | 20,134 | 1.00 |  |  |  |  |
|  | Privately rented | 5,776 | 1.35 (1.19-1.52) | <0.001 | 2,510 | 1.38 (1.25-1.52) | <0.001 |  |  |  |
|  | Provided housing | 1,018 | 1.29 (1.00-1.67) | 0.051 | 420 | 0.97 (0.76-1.23) | 0.777 |  |  |  |
|  | Publically subsidized | 1,611 | 1.31 (1.09-1.59) | 0.005 | 1,071 | 1.32 (1.15-1.51) | <0.001 |  |  |  |
|  | Rented rooms | 1,225 | 1.56 (1.26-1.93) | <0.001 | 548 | 1.32 (1.09-1.60) | 0.004 |  |  |  |

CI, confidence interval; OR, odds ratio.

^a^ Adjusted for age (per 5-year increase), gender, marital status, family size, smoking status, chronic medical conditions, and socioeconomic status (SES) factors (i.e., education, equivalent household expenditures, and occupation).

^b^ Adjusted for age, marital status, family size, smoking status, chronic medical conditions, and SES factors.

^c^ Adjusted for age, gender, marital status, family size, smoking status, and SES factors. Missing data for 898 participants.
