# Supplementary material for: Association between housing tenure and self-rated health in Japan: Findings from a nationwide cross-sectional survey
Source: PLoS One. 2019 Nov 14;14(11):e0224821. doi: 10.1371/journal.pone.0224821 (PMC6855483; doi:10.1371/journal.pone.0224821)
Supplement: S4 Table — (DOCX) [file pone.0224821.s004.docx]

**S4 Table.** Adjusted odds ratios for poor self-rated health based on stratified analyses by socioeconomic status factors

|  |  | n | OR (95% CI) | n | OR (95% CI) | n | OR (95% CI) | n | OR (95% CI) |
| --- | --- | --- | --- | --- | --- | --- | --- | --- | --- |
| Education^a^ | | University (n = 23,027) | | High school (n = 23,223) | | Junior high school (n = 8,192) | |  |  |
|  | Owner-occupied | 16,650 | 1.00 | 18,146 | 1.00 | 6,553 | 1.00 |  |  |
|  | Privately rented | 3,987 | ***1.27 (1.11-1.44)*** | 2,831 | ***1.43 (1.26-1.61)*** | 874 | ***1.49 (1.23-1.79)*** |  |  |
|  | Provided housing | 876 | 1.02 (0.79-1.30) | 424 | 0.97 (0.69-1.34) | 65 | 1.72 (0.92-3.22) |  |  |
|  | Publically subsidized | 755 | 1.23 (0.98-1.54) | 1,159 | ***1.31 (1.10-1.55)*** | 484 | ***1.52 (1.21-1.92)*** |  |  |
|  | Rented rooms | 759 | 1.18 (0.92-1.52) | 663 | ***1.47 (1.17-1.84)*** | 216 | ***1.60 (1.15-2.24)*** |  |  |
| EHE^b^ | | High (n = 17,508) | | Moderate (n = 19,342) | | Low (n = 20,512) | |  |  |
|  | Owner-occupied | 13,186 | 1.00 | 14,354 | 1.00 | 15,922 | 1.00 |  |  |
|  | Privately rented | 2,187 | ***1.24 (1.06-1.44)*** | 3,094 | ***1.43 (1.26-1.63)*** | 2,887 | ***1.37 (1.20-1.55)*** |  |  |
|  | Provided housing | 476 | 1.25 (0.90-1.73) | 486 | 1.10 (0.82-1.49) | 446 | 1.15 (0.85-1.55) |  |  |
|  | Publically subsidized | 893 | ***1.47 (1.21-1.77)*** | 880 | ***1.42 (1.18-1.72)*** | 842 | 1.04 (0.84-1.28) |  |  |
|  | Rented rooms | 766 | 1.22 (0.97-1.54) | 528 | ***1.48 (1.14-1.91)*** | 415 | ***1.47 (1.11-1.94)*** |  |  |
| Occupation^c^ | | Upper non-manual (n = 11,606) | | Lower non-manual (n = 14,844) | | Manual (n = 8,808) | | Non-working (n = 20,465) | |
|  | Owner-occupied | 8,475 | 1.00 | 10,726 | 1.00 | 6,732 | 1.00 | 16,299 | 1.00 |
|  | Privately rented | 1,893 | 1.13 (0.94-1.37) | 2,559 | ***1.29 (1.11-1.51)*** | 1,157 | 1.19 (0.95-1.48) | 2,284 | ***1.55 (1.38-1.76)*** |
|  | Provided housing | 515 | 1.16 (0.85-1.58) | 341 | 0.96 (0.65-1.41) | 200 | 0.63 (0.34-1.17) | 339 | 1.34 (0.97-1.84) |
|  | Publically subsidized | 381 | 0.84 (0.58-1.23) | 723 | 1.18 (0.92-1.50) | 440 | ***1.41 (1.05-1.89)*** | 1,002 | ***1.49 (1.27-1.74)*** |
|  | Rented rooms | 342 | 1.14 (0.79-1.64) | 495 | ***1.58 (1.19-2.10)*** | 279 | 1.06 (0.71-1.60) | 541 | ***1.45 (1.16-1.82)*** |

CI, confidence interval; EHE, Equivalent household expenditures; OR, odds ratio. Results in bold italic indicate *P* <0.05.

^a^ Adjusted for demographic factors (i.e., age, gender, marital status, family size, smoking status, and chronic medical conditions), EHE, and occupation. Missing data for 5,342 participants.

^b^ Adjusted for demographic factors, education, and occupation. Missing data for 2,422 participants.

^c^ Adjusted for demographic factors, education, and EHE. Missing data for 4,061 participants.
